# The Preventive Value of Acupoint Sensitization for Patients with Stable Angina Pectoris: A Randomized, Double-Blind, Positive-Controlled, Multicentre Trial

**DOI:** 10.1155/2021/7228033

**Published:** 2021-11-02

**Authors:** Shourui Huang, Ling Li, Jiali Liu, Xiujuan Li, Qingyang Shi, Ying Li, Yanping Liu, Mingxiu Li, Li Ma, Liang Ning, Xiaoyang Liao, Xihui Ying, Weiye Cai, Fuyu Yang, Tengfei Wang, Ru Guo, Weijie Ma, Wenzhu Chen, Jin Chen, Xin Sun

**Affiliations:** ^1^Chinese Evidence-based Medicine Centre, West China Hospital, Sichuan University, Chengdu 610041, China; ^2^School of Acupuncture and Massage, The Third Affiliated Hospital, Chengdu University of Traditional Chinese Medicine, Chengdu 610075, China; ^3^Graduate School of Chengdu University of Traditional Chinese Medicine, Chengdu 610036, China; ^4^Department of Cardiology, Xindu District Hospital of Traditional Chinese Medicine, Chengdu 610500, China; ^5^Department of Geriatrics, Sichuan Second Hospital of Traditional Chinese Medicine, Chengdu 610031, China; ^6^Department of Urology, West China Hospital, Sichuan University, Chengdu 610041, China; ^7^Department of Cardiology, No. 363 Hospital, Chengdu 610041, China; ^8^International Medical Centre, General Practice Unit, West China Hospital, Sichuan University, Chengdu 610041, China; ^9^Department of Traditional Chinese Medicine, The First People's Hospital of Longquanyi District, Chengdu 610041, China; ^10^Department of Traditional Chinese Medicine, No. 363 Hospital, Chengdu 610041, China; ^11^Department of Blood Transfusion, The First People's Hospital of Longquanyi District, Chengdu 610041, China

## Abstract

**Background:**

Acupoint sensitization is considered an important factor in the efficacy of acupoint therapy. This study aimed to evaluate the efficacy of acupressure in the prevention of stable angina pectoris using acupoints with different pressure-pain sensitivities.

**Methods:**

A total of 202 patients were enrolled and randomly assigned to a high-sensitivity group (HSG) (*n* = 109) in which patients received acupressure at the five acupoints with the highest sensitivity to pain and a low-sensitivity group (LSG) (*n* = 93) in which patients received acupressure at the five acupoints with the lowest sensitivity to pain. The duration of acupressure treatment was 4 weeks, and the patients were evaluated at baseline, week 4, and week 8. The primary outcome was a change in the frequency of angina attacks from baseline. The secondary outcomes included nitroglycerin consumption, the Canadian Cardiovascular Society classification, and the Seattle Angina Questionnaire score. Adverse events such as bleeding and subcutaneous haemorrhage were recorded in both groups.

**Results:**

The effect of acupressure compared with baseline on the prevention of angina pectoris in HSG was better than that in LSG at week 4 (incidence rate ratio (IRR): 0.691 and 95% confidence interval (CI): [0.569, 0.839]) and week 8 (IRR: 0.692 and 95% CI: [0.569, 0.839]). No significant difference between groups was found in the frequency of nitroglycerin consumption at week 4 (odds ratio (OR) = 0.863 and 95% CI: [0.147, 5.077]) or week 8 (OR = 1.426 and 95% CI: [0.211, 9.661]). Two themes in the questionnaire showed significantly different changes from baseline between the two groups. Scores on the angina frequency (AF) subscale had changed more from the baseline in the HSG at week 8 than in the LSG (mean difference (MD) = 3.807 and 95% CI: [0.673, 6.942]). Scores on the treatment satisfaction (TS) subscale had also changed more in the HSG than in the LSG at week 4 (MD = 3.651 and 95% CI: [0.327, 7.327]) and week 8 (MD = 4.220 and 95% CI: [0.347, 7.346]). One patient in the LSG reported bruising at the acupoint. No unexpected safety problems arose.

**Conclusions:**

This study showed that acupressure at acupoints with high sensitivity to pain may effectively reduce the frequency of stable angina pectoris episodes. This trial is registered with NCT03975140.

## 1. Introduction

Stable angina pectoris (SAP) is a clinical syndrome with discomfort in the anterior region of the chest as the main clinical manifestation and is the most common symptom of coronary heart disease (CHD) [1]. An attack of SAP not only limits the patient's mobility and reduces their quality of life but also imposes a serious burden on the society and economy [2]. Although clinical practice includes significant collective experience related to the treatment of SAP, the treatments have limitations. For example, long-term medication can adversely affect other organs and cause drug resistance. In addition, invasive percutaneous coronary intervention and coronary artery bypass grafting are associated with various adverse events and high risk of postoperative bleeding. Therefore, the key to improving the therapeutic effect of SAP is to find an appropriate adjuvant treatment with the potential to reduce the frequency of SAP and patients' drug dependence and avoid aggravation of the disease.

Traditional Chinese medicine (TCM) clinicians often use acupuncture or acupressure as adjunctive therapies for antianginal treatment. Acupuncture has been widely used in clinical practice around the world for thousands of years because of its convenience, safety, and effectiveness [3]. It is generally believed that acupoints, where acupuncture works on the human body surface, are the basis of acupuncture treatment effectiveness [4]. Modern studies of TCM show that acupoints considered to be associated with the viscera often remain in a “resting” state during periods of good health and become “active” in pathology [5, 6]. This transformation or switch between states is known as acupoint sensitization. Early studies found that this phenomenon was often accompanied by “abnormal sensitization” near specific acupoints, mainly manifested as increased sensitivity to pain or heat, discomfort, and striated subcutaneous nodules [7]. The occurrence and development of a disease may lead to changes in the degree of sensitization. Published functional and physiological experiments have suggested that minor external stimulation at sensitized acupoints may trigger clear physical reactions in patients [8]. The degree of acupoint sensitization may be related to the severity of the disease, and early intervention on sensitized acupoints may improve the effectiveness of acupuncture treatment.

In recent years, the theory of acupoint sensitization has been widely applied in China. Previous studies have found that the infrared radiation spectra of PC6, PC8, HT7, LU9, and LR3 and other acupoints in patients with CHD were significantly lower than those in healthy people [9–13], demonstrating acupoint sensitization in patients with CHD. Therefore, the present study aimed to explore whether acupoint sensitization has scope for alleviation of viscera disease by conducting a randomized controlled trial to compare the effects of stimulation at acupoints with different sensitivities on the prevention of angina pectoris.

## 2. Materials and Methods

### 2.1. Study Design and Population

This study was a multicentre randomized controlled trial in which outpatients and inpatients with SAP were recruited from the departments of acupuncture and cardiology in six different clinical units in Chengdu, China. Patients were enrolled in this study from June 12, 2019, to November 11, 2020. Diagnosis of SAP was in accordance with the guideline for the diagnosis and treatment of stable coronary artery disease issued by the Chinese Society of Cardiology in 2018 [14] and with reference to the diagnostic and classification criteria of stable angina pectoris designated by the American Heart Association [15].

Participants were enrolled if they fulfilled the following criteria: diagnosed with stable angina pectoris and symptoms of chest pain; duration of disease over 3 months, with at least two angina attacks in the past month; aged between 30 and 80 years; and voluntary participation and signed informed consent. Patients with any of the following conditions were excluded: intellectual disability; contraindications or inability to complete the acupoint sensitization test; cardiovascular, digestive, urinary, respiratory, blood, nerve, endocrine, or other serious primary diseases; bleeding or allergy disorders; abnormal skin or peripheral nerve sensation, abnormal pain sensation, and skin ulceration at the sensitization detection site; and unsatisfactory clinical treatment of hypertension or diabetes.

The protocol was approved by the ethics review board of the West China Hospital, Sichuan University (No. 286, in 2019) and was published on clinicaltrials.gov (NCT03975140). Patients provided handwritten informed consent.

### 2.2. Randomization and Blinding

Eligible patients were randomly assigned with equal probability to groups via a central randomization system. An independent researcher who was not involved in the intervention, data collection, or outcome assessment was charged with patient allocation. Patients and research personnel (apart from the statistician, including intervention providers and outcome assessors) were blinded to group allocation throughout the study.

### 2.3. Intervention

All enrolled patients were required to take an acupoint sensitization detection (ASD) test before and after the intervention. Acupuncturists operated the von Frey detector to measure pressure-pain thresholds (PPT) of 12 disease-related acupoints determined from the previous literature and expert opinions [16, 17]. The final PPTs of the 12 acupoints of each patient in this study were ordered independently by their threshold. The five acupoints with the highest thresholds were selected to be used in the low-sensitivity group (LSG), and the five acupoints with the lowest thresholds were selected to be used in the high-sensitivity group (HSG). The process and details of ASD are shown in Supplementary Files S1-S2. In both groups, standard acupressure was applied by professional acupuncturists, and the frequency of treatment was three times per week for 4 weeks. Patients who missed three or more sessions were excluded. Further details of standard acupressure are shown in Supplementary File S3.

All patients received conventional antianginal therapy during the 8-week study period. The choice of pharmacologic treatment was individualized, with consideration of each patient's coexisting conditions and adverse effects to the medications. In the case of an acute angina attack, patients were allowed to use the following medications for emergency medication to relieve discomfort: nitroglycerin and Suxiao Jiuxin Wan (a TCM product). All patients were prohibited from taking nitroglycerin and Suxiao Jiuxin Wan for preventive purposes during the study period.

### 2.4. Follow-Up and Outcomes

The study period was 8 weeks in total, including treatment for 4 weeks and follow-up for 4 weeks. Blinded evaluators recorded results in customized case report forms at week 0, week 4, and week 8, respectively. The primary outcome was the change in the frequency of angina attacks from baseline. Secondary outcomes included nitroglycerin consumption for rescue medication, Canadian Cardiovascular Society classification (CCS), Seattle Angina Questionnaire (SAQ) data, and change in mean PPT at the acupoints from baseline to week 4. Adverse events which could be related to acupressure, including bleeding, subcutaneous haemorrhage, hematoma, fainting, serious pain, and local infection, were recorded in both groups during this study.

### 2.5. Sample Size

The frequency of angina attacks (number within 4 weeks) was used as the primary outcome index to estimate the sample size. Based on the relevant literature, the values were as follows: *β* = 0.1, *α* = 0.05, the mean difference (MD) between the treatment group and the control group was 4, and the standard deviation was 7. The required sample size of each group was estimated to be 65 cases. With an estimated attrition rate of about 15%, the sample size of this study was determined to be 80 cases in each of the two groups, so the minimum sample size was 160 cases.

### 2.6. Statistical Analysis

Percentage or frequency were used for statistical descriptions of category data. Mean ± standard deviation was used to describe normally distributed continuous data. The main analyses were based on the modified intention-to-treat (ITT) principle, including participants with at least one measurement of outcomes. A Poisson regression mixed-effect model was established for the frequency of angina attacks. The incidence rate ratio (IRR) and its 95% confidence interval (CI) were used to evaluate the difference in efficacy between the groups. A mixed-effects model of ordinal data was established for the CCS classification of angina pectoris and the frequency of therapeutic nitroglycerin use, with between-group differences evaluated using odds ratio (OR) and its 95% CI. Different linear mixed-effects models were constructed for SAQ scores of different dimensions, and MD and 95% CI were used to evaluate the efficacy difference between groups. Risk factors of SAP suggested by guidelines [14], such as age, body mass index, and diabetes history, were analysed by post hoc stratification, and the potential influencing factors for the efficacy of different sensitized acupoints were explored.

Electronic data capture systems were used to store clinical information about the study subjects; Excel 2019 software was used for data export and management. *R* 4.0.4 (via *R* Studio) software was used for data cleaning, merging, and statistical analysis.

## 3. Results

### 3.1. Patient Characteristics at Baseline

**Figure 1:**
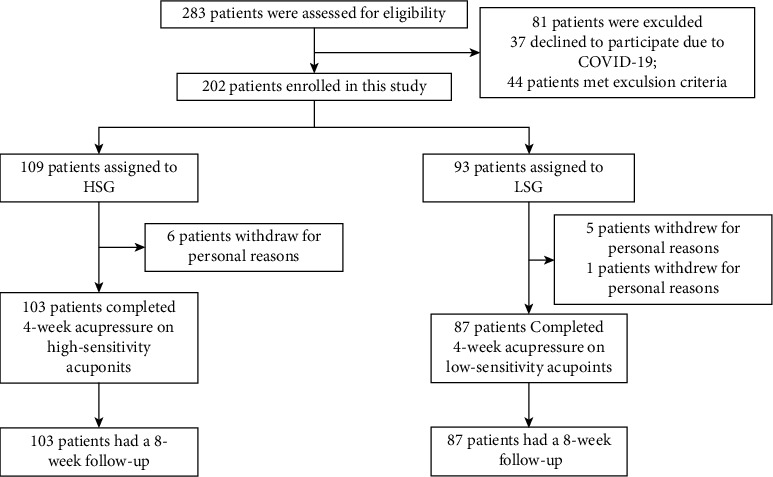
Flowchart of enrolment, randomization, and follow-up. HSG: high-sensitivity group; LSG: low-sensitivity group.

Between 12 June 2019 and 11 November 2020, 202 patients were enrolled in this study and randomly assigned to the HSG and LSG (HSG: 109 cases; LSG: 93 cases). Twelve patients withdrew from the treatment for personal reasons, leaving 190 cases who completed treatment and follow-up (Figure 1). Patients' characteristics are shown in Table 1. One hundred and eleven cases (54.95%) were women, and the average age was 66.13 ± 9.51 years; 53 patients (26.24%) had a history of regular smoking; 32 patients (15.84%) had a history of regular drinking; 23 patients (11.39%) had a family history of CHD; 88 patients (43.56%) had hypertension. Forty-two patients (20.79%) had diabetes, and 22 patients (10.89%) had previous experience of drug allergy; the average number of angina pectoris episodes in the recent four weeks at baseline was 5.96 ± 5.24. None of the characteristics differed significantly between the two groups (*P* > 0.05).

All 202 patients underwent ASD at baseline. The baseline PPT at each acupoint of patients was comparable between the groups (*P* > 0.05). Patients in the HSG chose HT3, HT7, RN17, PC6, HT6, and PC3 and other acupoints more frequently, while those in the LSG chose BL15, BL16, BL14, and RN14 more frequently. The distribution of acupoint selection frequency and the average PPT of 12 acupoints at baseline are both shown in Supplementary Files S4-S5.

202 cases underwent therapy in this study, of which 12 patients withdrew for personal reasons during the treatment and were not included in the subsequent efficacy analysis. The baseline characteristics of excluded patients are detailed in Supplementary File S6.

### 3.2. Primary Efficacy Outcome

**Figure 2:**
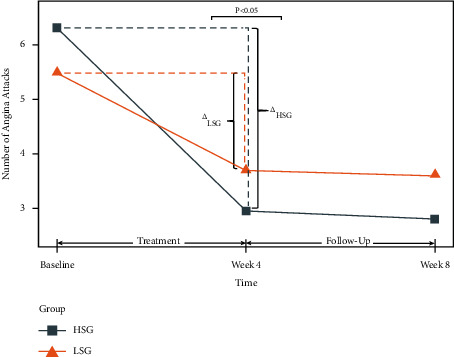
Change in the frequency of angina attacks during the study. HSG: high-sensitivity group; LSG: low-sensitivity group; Δ_HSG_: changes of the number of angina attacks from baseline to week 4 in the high-sensitivity group; and Δ_LSG_: changes of the number of angina attacks from baseline to week 4 in the low-sensitivity group.

The mixed-effects model of angina attacks based on Poisson regression is shown in Table 2. At week 4 and at week 8, the risk of angina attack in the HSG was significantly lower than that in the LSG (IRR = 0.691 and 95% CI: [0.569, 0.839] in week 4; IRR = 0.692 and 95% CI: [0.568, 0.841] in week 8). The frequency of angina attacks was reduced from baseline in the HSG significantly more than in the LSG at week 4 (*P* < 0.001) and week 8 (*P*=0.003) (Figure 2). The 168 patients who received 12 sessions of treatment, and were, therefore, adherent to treatment, were selected for sensitivity analysis with respect to the primary efficacy outcome. At weeks 4 and 8, the frequency of angina attacks in the HSG was significantly lower than that in the LSG (IRR = 0.710 and 95% CI: [0.582, 0.867] in week 4; IRR = 0.677 and 95% CI: [0.553, 0.829] in week 8).

**Figure 3:**
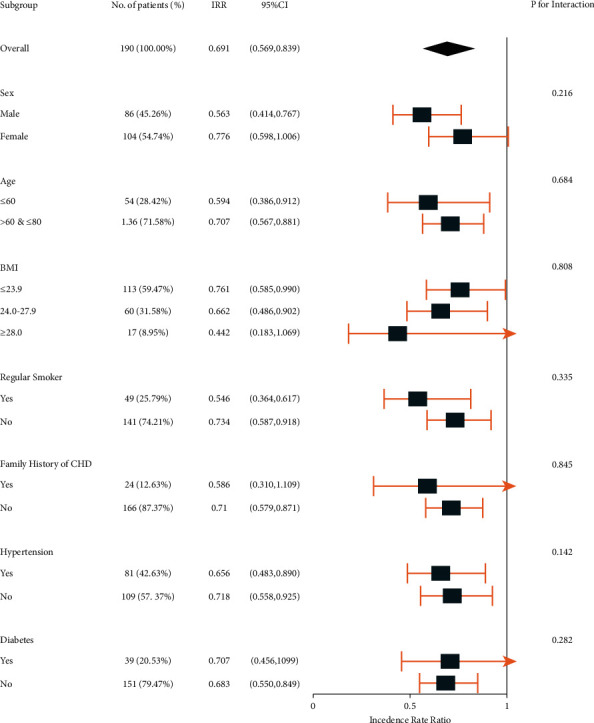
Subgroup analysis of associations between angina attack and intervention. IRR: incidence rate ratio; CI: confidence interval; BMI: body mass index; and CHD: coronary heart disease.

We examined the associations between the frequency of angina attacks and different interventions according to the risk factors suggested by SAP guidelines. The associations were statistically similar between subgroups within each risk factor, as shown in Figure 3.

### 3.3. Secondary Outcome

A mixed-effects model based on ordinal logistic regression was used to compare nitroglycerin consumption at different study periods between groups. At week 4 and week 8, the nitroglycerin consumption difference from baseline was similar in the HSG and LSG (OR = 0.863 and 95% CI: [0.147, 5.077] in week 4; OR = 1.426 and 95% CI: [0.211, 9.661] in week 8).

At week 8, the SAQ AF subscale score difference from baseline was higher in the HSG than in the LSG (MD = 3.807 and 95% CI: [0.673, 6.942]). At weeks 4 and 8, the TS subscale score differences from baseline were higher in the HSG than in the LSG (MD = 3.651, 95% CI [0.327, 7.327] in week 4; MD = 4.220 and 95% CI: [0.347, 7.346] in week 8). On the remaining subscales, the difference from baseline at each period was similar in the two groups. No significant difference between groups was found in the CCS classification at week 4 (OR = 0.805 and 95% CI: [0.201, 3.222]) or week 8 (OR = 1.542 and 95% CI: [0.376, 6.334]). The analyses reported above are shown in Supplementary Files S7-S8.

**Figure 4:**
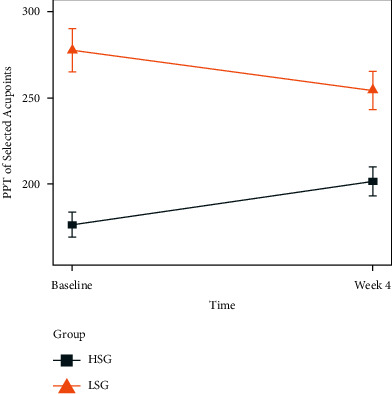
Change of PPT between groups from baseline to week 4. HSG: high-sensitivity group; LSG: low-sensitivity group; PPT: pressure-pain threshold; and error bars indicate the standard error of the mean.

Compared with baseline, average changes of PPT at acupoints after treatment are shown in Figure 4. It can be seen that, after treatment, the mean PPT in the HSG increased from 175.935 g/N to 201.232 g/N (*P* < 0.05), and the mean PPT in the LSG decreased from 277.656 g/N to 254.279 g/N (*P* < 0.05).

### 3.4. Safety Outcome

Only one patient in the LSG reported an intervention-related adverse event, which consisted of bruising at the acupoint. The patient declined to continue and chose to withdraw from the treatment. No unexpected safety problems were encountered in the study.

## 4. Discussion

To our knowledge, this study is the first multicentre randomized controlled trial to explore the relationship between SAP and acupoint pain sensitivity, using an objective detection method and high methodological quality. This study explored and verified the relationship between acupoint sensitization and disease severity and found that the stimulation of acupoints with high pain sensitivity has potential as an adjuvant antianginal treatment which may be beneficial to patients.

This study found that compared with baseline, the frequency of angina attacks at week 4 was significantly lower in the HSG than in the LSG and that nitroglycerin consumption was significantly reduced in both groups at week 4 and 8. These findings indicate that there may be a dose-response relationship between acupoint sensitivity and disease severity, consistent with published mechanistic studies. For example, Wang et al. [18] found that stimulating pain-sensitive acupoints in rats may regulate visceral function to achieve an analgesic effect. Our findings suggest that stimulation of pain-sensitive acupoints may help to prevent SAP episodes.

In an animal model of acute intestinal mucosal loss, Shi et al. [19, 20] found that acupoints show subcutaneous mast cell recruitment and degranulation, which may excite neurons and stimulate skin cells to synthesize and secrete cytokines related to immunity. These cytokines could induce sympathetic generation in the corresponding segment of the ganglion to promote the self-healing of internal organs [11, 21]. Therefore, appropriate stimulation of sensitized acupoints may theoretically mobilize the body's self-healing function. However, in the present study, no significant effect was found in the secondary outcomes, including CCS grade and SAQ scores. This may reflect the fact that most (190) of the patients included in this study had a baseline number of angina attacks of 5.96 ± 5.24, and 168 (88.42%) patients were at CCS grade I or II, with moderate SAP, and with quality of life not severely affected by angina pectoris [22].

In this study, the von Frey detector was used to assess PPT at disease-related acupoints. Published observational studies have suggested that PPTs at acupoints are decreased in most patients and decrease further with disease severity [23–26]. In recent years, experimental studies have found that histamine, 5-hydroxytryptamine, prostaglandin, bradykinin, substance-P, and nerve growth factor were highly concentrated in the subcutaneous tissue around the sensitive acupoints in animal models [27, 28], and the accumulation of these substances in the local skin was considered to be the material basis of abnormal sensitivity of acupoints [6, 16]. However, the present study found that, for both groups, although posttreatment SAP was alleviated compared with baseline, PPT changes differed between groups, with increases in HSG and decreases in LSG. To date, no research has reported that PPT of acupoints decreases during disease recovery. This study only evaluated PPT twice, before and after the intervention, and may have missed any dynamic change in the acupoint pain threshold. However, the findings suggest that acupoint sensitization may not vary linearly with disease severity. Owing to the instability of mechanistic studies [29], further dynamic monitoring technology is needed to explain the complex process of acupoint sensitization [30].

This study is the largest multicentre randomized controlled trial to date on SAP under the guidance of acupoint sensitization theory. With a strict design and objective intervention, this study has great significance in demonstrating the preventive value of acupoint sensitization and the potential to improve the clinical efficacy of traditional acupoint therapy.

There were two limitations in this study. First, there was no sham acupressure group, limiting the extrapolation of the conclusion. Second, only one form of acupoint sensitization, pain sensitivity, was studied. Since the measurement of the pain threshold of patients is readily affected by subjective patient-related factors, the pain threshold varies greatly between patients. Follow-up studies should also focus on other forms of acupoint sensitization, such as photosensitivity and thermosensitivity.

## 5. Conclusions

The frequency of angina pectoris attacks in patients who received acupressure at acupoints with high pain sensitivity was less than that in patients who received the acupressure at acupoints with low pain sensitivity. Acupoint sensitization phenomena have clinical value in the adjuvant treatment of stable angina pectoris.

## Figures and Tables

**Table 1:** Baseline characteristics of included patients.

Characteristic^1^	Group		
	LSG (*N* = 93)	HSG (*N* = 109)	Overall (*N* = 202)
**Age (mean** **±** **SD)**	66.22 ± 10.02	66.06 ± 9.11	66.13 ± 9.51
**Sex (female)**	48.0 (51.6%)	63.0 (57.8%)	111 (55.0%)
**Marital status (married)**	86.0 (92.5%)	101.0 (92.7%)	187 (92.6%)
**BMI (Mean** **±** **SD)**	23.34 ± 3.34	23.14 ± 3.17	23.23 ± 3.24

Education			
Basic to junior high school	73 (78.5%)	91 (83.5%)	164 (81.2%)
High school and above	20 (21.5%)	18 (16.5%)	38 (18.8%)
**Regular smoker (yes)**	27 (29.0%)	26 (23.9%)	53 (26.2%)
**Regular drinker (yes)**	16 (17.2%)	16 (14.7%)	32 (15.8%)
**Previous drug allergies (yes)**	8 (8.6%)	14 (12.8%)	22 (10.9%)
**CHD family history (yes)**	9 (9.7%)	14 (12.8%)	23 (11.4%)
**Hypertension (yes)**	39 (41.9%)	49 (45.0%)	88 (43.6%)
**Diabetes (yes)**	17 (18.3%)	25 (22.9%)	42 (20.8%)
**Aspirin (yes)**	35 (37.6%)	40 (36.7%)	75 (37.1%)
**Clopidogrel (yes)**	19 (20.4%)	13 (11.9%)	32 (15.8%)
***β*-Blockers (yes)**	17 (18.3%)	27 (24.8%)	44 (21.8%)
**Statins (yes)**	51 (54.8%)	53 (48.6%)	104 (51.5%)
**Angiotensin inhibitor (yes)**	4 (4.3%)	4 (3.7%)	8 (4.0%)
**Calcium antagonists (yes)**	24 (25.8%)	30 (27.5%)	54 (26.7%)
**Frequency of angina attacks (mean** **±** **SD)**	5.52 ± 4.48	6.34 ± 5.81	5.96 ± 5.24

Nitroglycerin consumption			
None	62 (66.7%)	76 (69.7%)	138 (68.3%)
<3 times/week	23 (24.7%)	27 (24.8%)	50 (24.8%)
3–12 times/week	7 (7.5%)	6 (5.5%)	13 (6.4%)
≥12 times/week	1 (1.1%)	0 (0.0%)	1 (0.5%)

CCS grade			
Level I	45 (48.4%)	49 (45.0%)	94 (46.5%)
Level II	40 (43.0%)	45 (41.3%)	85 (42.1%)
Level III	7 (7.5%)	14 (12.8%)	21 (10.4%)
Level IV	1 (1.1%)	1 (0.9%)	2 (1.00%)

SAQ score			
Physical limitations	52.38 ± 18.81	51.44 ± 19.36	51.87 ± 19.06
Angina stability	51.62 ± 20.12	54.82 ± 21.91	53.34 ± 21.12
Angina frequency	80.08 ± 14.37	77.50 ± 16.53	78.69 ± 15.59
Treatment satisfaction	68.75 ± 20.54	68.81 ± 21.72	68.78 ± 21.14
Quality of life	58.87 ± 24.57	62.61 ± 23.20	60.89 ± 23.85

SD: standard deviation; CHD: coronary heart disease; CCS: Canadian Cardiovascular Society; SAQ: Seattle Angina Questionnaire; ^1^mean ± SD or frequency (%); ^2^Wilcoxon rank sum test; Pearson's chi-squared test; Fisher's exact test.

**Table 2:** Between-group comparison of the frequency of angina attacks at different study phases.

Study phase	Frequency of attacks (mean ± SD)		Adjusted model^1^	
	HSG	LSG	HSG vs. LSG	
			IRR (95% CI)^2^	*P* value
Baseline	6.31 ± 5.75	5.49 ± 4.55	—	—
Week 4	2.96 ± 3.42	3.69 ± 3.88	0.691 (0.569, 0.839)	0.002
Week 8	2.81 ± 3.48	3.60 ± 3.68	0.692 (0.568, 0.841)	0.002

HSG: high-sensitivity group; LSG: low-sensitivity group; IRR: incidence rate ratio; CI: confidence interval. ^1^Adjusted model was constructed by a Poisson mixed model with treatment, time, and interaction between treatment and time as the fixed effects and centre and individuals as the random effects. ^2^Compared with baseline, the difference of change of angina attacks between groups was reflected by the incidence rate ratio.

## Data Availability

The datasets supporting the conclusion of this study are not publicly available due to possible violation of ethical review requirements but are available from the corresponding author on reasonable request.

## Abbreviations

SAP: Stable angina pectoris
CHD: Coronary heart disease
TCM: Traditional Chinese medicine
ASD: Acupoint sensitization detection
PPT: Pressure-pain thresholds
HSG: High-sensitivity group
LSG: Low-sensitivity group
CCS: Canadian Cardiovascular Society
SAQ: Seattle Angina Questionnaire
MD: Mean difference
ITT: Intention to treat
IRR: Incidence rate ratio
CI: Confidence interval
OR: Odds ratio.

## Supplementary Materials

Supplementary Materials CONSORT 2010 checklist. This section reported the CONSORT checklist of this study. S1: acupoint locations. This section introduced the locations of acupoints in the human body of used in this study and includes Figures s1–s2. S2: Acupoint Sensitization Detection (ASD). This section describes the measurement of pain threshold at acupoints at baseline and the fourth week. S3: standard acupressure. This section explains the acupuncturists' treatment approach for the two groups. S4: frequency of acupoint selection. This section describes the frequency of acupoint selection for patients in different groups based on pressure-pain thresholds and includes Figures s3–s4. S5: PPT of 12 acupoints at baseline. This section describes the distribution of pressure-pain threshold of 12 acupoints of all patients in this study and includes Figure s5. S6: baseline characteristics of excluded patients. This section shows the baseline characteristics of excluded patients in this study and includes Table s1. S7: mixed-effects model of SAQ scores of patients at different study periods. This section includes the results of the mixed-effect model analysis of the scores on the Seattle Angina Questionnaire of patients at different study periods, shown in Table s2. S8: mixed-effects models based on the ordinal regression of CCS grade. This section includes results of the mixed-effects model analysis of the Canadian Cardiovascular Society classification of patients in different study periods, shown in Table s3.
