# Supplementary material for: The Preventive Value of Acupoint Sensitization for Patients with Stable Angina Pectoris: A Randomized, Double-Blind, Positive-Controlled, Multicentre Trial
Source: Evid Based Complement Alternat Med. 2021 Nov 2;2021:7228033. doi: 10.1155/2021/7228033 (PMC8577890; doi:10.1155/2021/7228033)
Supplement: Supplementary Materials — CONSORT 2010 checklist. This section reported the CONSORT checklist of this study. S1: acupoint locations. This section introduced the locations of acupoints in the human body of used in this study and includes Figures s1–s2. S2: Acupoint Sensitization Detection (ASD). This section describes the measurement of pain threshold at acupoints at baseline and the fourth week. S3: standard acupressure. This section explains the acupuncturists' treatment approach for the two groups. S4: frequency of acupoint selection. This section describes the frequency of acupoint selection for patients in different groups based on pressure-pain thresholds and includes Figures s3–s4. S5: PPT of 12 acupoints at baseline. This section describes the distribution of pressure-pain threshold of 12 acupoints of all patients in this study and includes Figure s5. S6: baseline characteristics of excluded patients. This section shows the baseline characteristics of excluded patients in this study and includes Table s1. S7: mixed-effects model of SAQ scores of patients at different study periods. This section includes the results of the mixed-effect model analysis of the scores on the Seattle Angina Questionnaire of patients at different study periods, shown in Table s2. S8: mixed-effects models based on the ordinal regression of CCS grade. This section includes results of the mixed-effects model analysis of the Canadian Cardiovascular Society classification of patients in different study periods, shown in Table s3. [file 7228033.f1.doc]

**Supplementary File**

**The preventive value of acupoint sensitization for patients with stable angina pectoris: a randomized, double-blind, positive-controlled, multi-centre trial**

Shourui Huang

E-mail: [531239462@qq.com](mailto:531239462@qq.com)

Co-Corresponding author: Jin Chen; [ebm_chenjin@126.com](mailto:ebm_chenjin@126.com);

Xin Sun; sunxin@wchscu.cn;

**CONTENTS**

[**CONSORT 2010 checklist** 1](#__RefHeading___Toc85219173)

[**S1 Locations of acupoints needed to be detected** 4](#__RefHeading___Toc85219174)

[**S2 Acupoint Sensitization Detection (ASD)** 5](#__RefHeading___Toc85219175)

[**S3 Standard Acupressure** 7](#__RefHeading___Toc85219176)

[**S4 Frequency of Acupoints Selection** 8](#__RefHeading___Toc85219177)

[**S5 PPT of 12 acupoints at baseline** 9](#__RefHeading___Toc85219178)

[**S6 Baseline characteristics of excluded patients** 10](#__RefHeading___Toc85219179)

[**S7 Mixed effect model of SAQ score of patients in different periods** 12](#__RefHeading___Toc85219180)

[**S8 Mixed effect models based on ordinal regression of CCS grade** 13](#__RefHeading___Toc85219181)

**CONSORT 2010 checklist**

| Section/Topic | Item No | Checklist item | Reported on page No |
| --- | --- | --- | --- |
| Title and abstract | | | |
|  | 1a | Identification as a randomized trial in the title | 1 |
|  | 1b | Structured summary of trial design, methods, results, and conclusions (for specific guidance see CONSORT for abstracts) | 3 |
| Introduction | | | |
| Background and objectives | 2a | Scientific background and explanation of rationale | 5 |
|  | 2b | Specific objectives or hypotheses | 6 |
| Methods | | | |
| Trial design | 3a | Description of trial design (such as parallel, factorial) including allocation ratio | 7 |
|  | 3b | Important changes to methods after trial commencement (such as eligibility criteria), with reasons | None |
| Participants | 4a | Eligibility criteria for participants | 7 |
|  | 4b | Settings and locations where the data were collected | 7 |
| Interventions | 5 | The interventions for each group with sufficient details to allow replication, including how and when they were actually administered | 7 |
| Outcomes | 6a | Completely defined pre-specified primary and secondary outcome measures, including how and when they were assessed | 8 |
|  | 6b | Any changes to trial outcomes after the trial commenced, with reasons | None |
| Sample size | 7a | How sample size was determined | 10 |
|  | 7b | When applicable, explanation of any interim analyses and stopping guidelines | None |
| Randomisation: |  |  |  |
| Sequence generation | 8a | Method used to generate the random allocation sequence | 7 |
|  | 8b | Type of randomisation; details of any restriction (such as blocking and block size) | 7 |
| Allocation concealment mechanism | 9 | Mechanism used to implement the random allocation sequence (such as sequentially numbered containers), describing any steps taken to conceal the sequence until interventions were assigned | 7 |
| Implementation | 10 | Who generated the random allocation sequence, who enrolled participants, and who assigned participants to interventions | 7 |
| Blinding | 11a | If done, who was blinded after assignment to interventions (for example, participants, care providers, those assessing outcomes) and how | 7 |
|  | 11b | If relevant, description of the similarity of interventions | None |
| Statistical methods | 12a | Statistical methods used to compare groups for primary and secondary outcomes | 9 |
|  | 12b | Methods for additional analyses, such as subgroup analyses and adjusted analyses | 9 |
| Results | | | |
| Participant flow (a diagram is strongly recommended) | 13a | For each group, the numbers of participants who were randomly assigned, received intended treatment, and were analysed for the primary outcome | 10 |
|  | 13b | For each group, losses and exclusions after randomisation, together with reasons | 10 |
| Recruitment | 14a | Dates defining the periods of recruitment and follow-up | 10 |
|  | 14b | Why the trial ended or was stopped | 10 |
| Baseline data | 15 | A table showing baseline demographic and clinical characteristics for each group | 10 |
| Numbers analysed | 16 | For each group, number of participants (denominator) included in each analysis and whether the analysis was by original assigned groups | 11 |
| Outcomes and estimation | 17a | For each primary and secondary outcome, results for each group, and the estimated effect size and its precision (such as 95% confidence interval) | 13 |
|  | 17b | For binary outcomes, presentation of both absolute and relative effect sizes is recommended | 13 |
| Ancillary analyses | 18 | Results of any other analyses performed, including subgroup analyses and adjusted analyses, distinguishing pre-specified from exploratory | 15 |
| Harms | 19 | All important harms or unintended effects in each group (for specific guidance see CONSORT for harms) | 17 |
| Discussion | | | |
| Limitations | 20 | Trial limitations, addressing sources of potential bias, imprecision, and, if relevant, multiplicity of analyses | 19 |
| Generalisability | 21 | Generalisability (external validity, applicability) of the trial findings | 17 |
| Interpretation | 22 | Interpretation consistent with results, balancing benefits and harms, and considering other relevant evidence | 17 |
| Other information | | |  |
| Registration | 23 | Registration number and name of trial registry | 20 |
| Protocol | 24 | Where the full trial protocol can be accessed, if available | 20 |
| Funding | 25 | Sources of funding and other support (such as supply of drugs), role of funders | 20 |

**S1** **Locations of acupoints needed to be detected**

According to the literature review and expert opinion, 12 acupoints to be detected were determined. Referring to the textbook standard of acupuncture and moxibustion published by the people's Health Publishing House in Beijing, the location of acupoints to be detected in this study is roughly shown in Figure S1-S2.

**
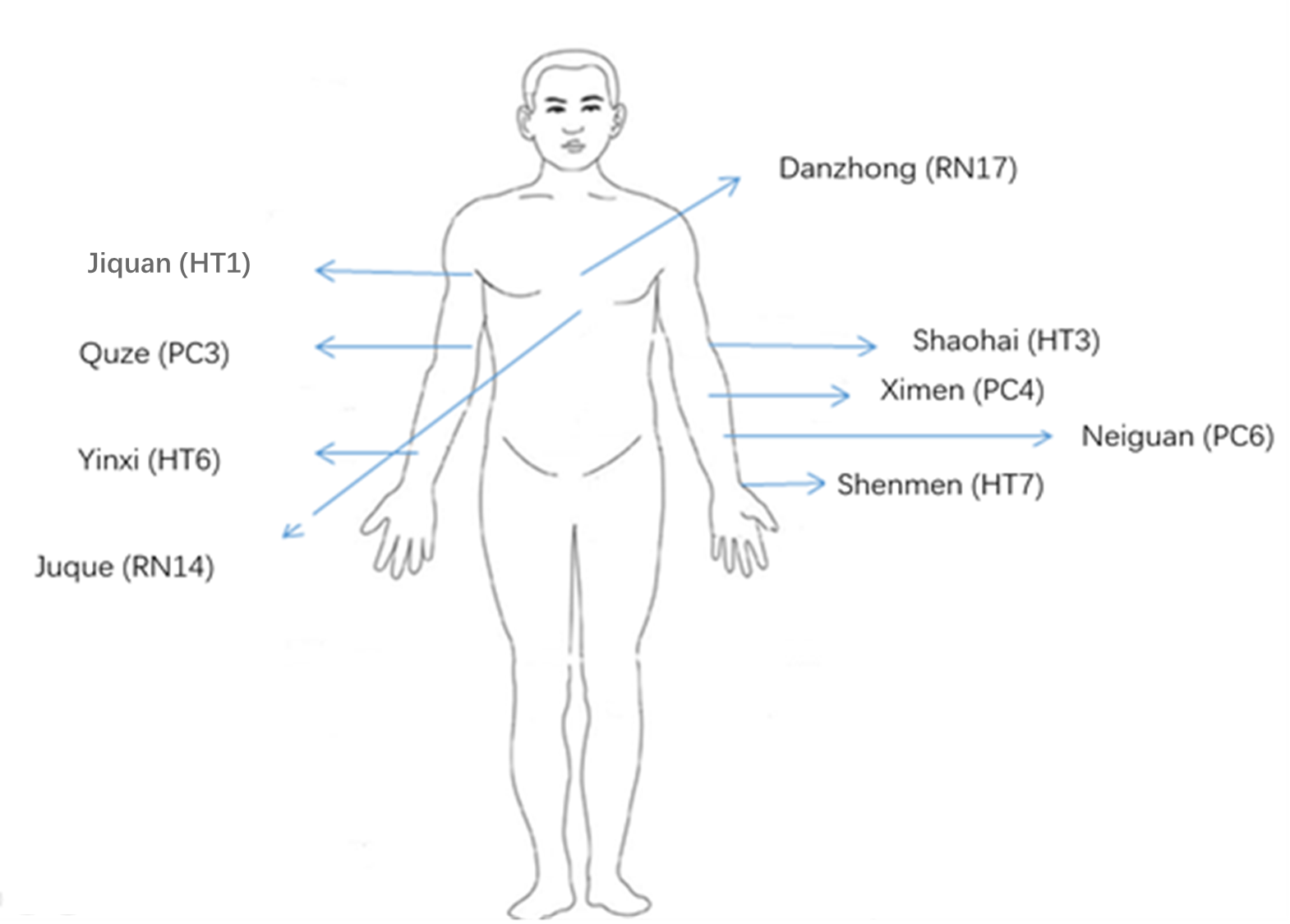
**

**Figure s1. General location of the acupoints of the upper limbs and chest**


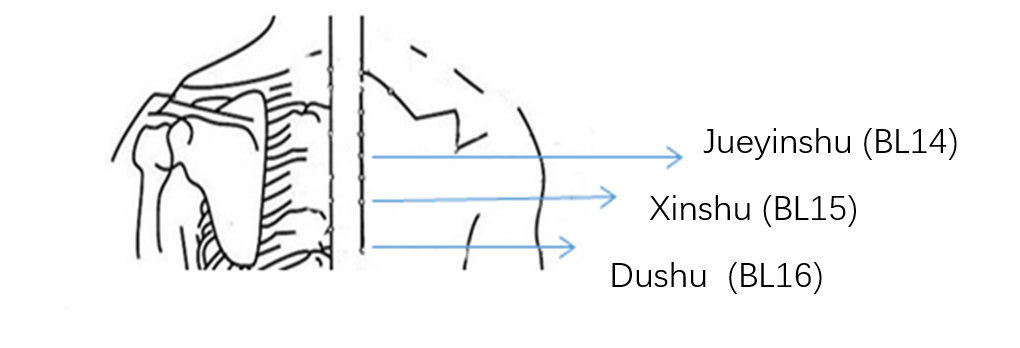


**Figure s2. General location of the acupoints of back**

**S2 Acupoint Sensitization Detection (ASD)**

All patients who met the inclusion and exclusion criteria were required to take Acupoint Sensitization Detection (ASD). Von Frey detector (IITC Inc., USA) was used to detect the Pressure-Pain Threshold (PPT) of left acupoints and peripheral palpation tenderness points. The PPT of acupoints could reflect the pain tolerance of acupoints. The operation process of ASD was as follows:

1. **Preparation**

After the patients were enrolled, the researchers need to inform the patients of the specific matters of acupoint sensitization detection in time. Before the work of acupoint sensitization detection, the researchers need to communicate with them patiently to eliminate the patients' tension. Set up an independent sensitized acupoint detection workshop to ensure the confidentiality and privacy of patients in the process of research. In addition, the staff need other medical staff to be present when performing acupoint sensitization detection for female patients.

1. **Calibration**

Lay the Von Frey electronic detector and display horizontally, press and hold the "on / off" key to start the machine. Use the "CLR" key to clear the current reading. When the display shows "0.0", use a calibrator with a weight of 5.3g to calibrate the result. After the result is stable, if the display shows that the result is within "5.3 ± 0.1g/n", it is considered that the instrument has been calibrated.

1. **Pilot operation on patient before the formal detection.**

An obtuse angle probe was installed on the Von Frey electronic detector. Before the formal detection, patients were trained to take stimulation on acupoints for few times. The reactions of the subjects were observed, and the subjects were instructed to report to the researchers immediately when the "acupuncture like" sensation appeared at the stimulated acupoints.

1. **Formal detection**

The acupuncturist with the qualification certificate of traditional Chinese medicine holds the detector and aims the probe needle at the acupoint to be tested. Another data recorder holds the display and presses the "CLR" and "Max" keys respectively before the acupuncturist puts down the needle each time to clear the current reading and display the maximum threshold value of the detection. Then the acupuncturist will put the probe needle perpendicular to the acupoint to be tested Measure the acupoints, apply force evenly, press and stimulate the acupoints. When the subject reported "acupuncture like" pain at the detected acupoint, the acupuncturist immediately removed the detector, and the data recorder independently read and recorded the data on the display screen.

1. **Recording and checking of the results.**

The sequence of acupoint detection is from the extremities to the armpits, and from the front chest to the back. In order to ensure the stability of the detection results, all the acupoints to be detected need to be detected twice. After the first detection of all acupoint pain threshold, the second detection is carried out to ensure that the detection interval of a single acupoint is 15-20 minutes. When the difference between the two readings of the same acupoint is less than or equal to 15g / N, the average of the two results is taken as the final pain threshold of the acupoint; if the two readings are more than 15g / N, the acupoint is detected for the third time, and the average of the two readings with the smallest difference among the three readings is taken as the final pain threshold of the acupoint.

**S3 Standard Acupressure**

According to the results of randomization and ASD at baseline, the standard acupoints provided in this study were determined, and acupuncturists with Chinese medicine qualification certificate performed standard acupoint pressing. Acupuncturist use the thumb to pressure on the surface of the target acupoint slowly apply force until patients feel the pressure appear acid, numbness, distension and other feeling known as "De Qi", and then continue to roll the target acupoint with the same force for a total of three times, finally the doctor release force to relax. Repeat the above action 2 minutes for a single target acupoint.

**S4 Frequency of Acupoints Selection**


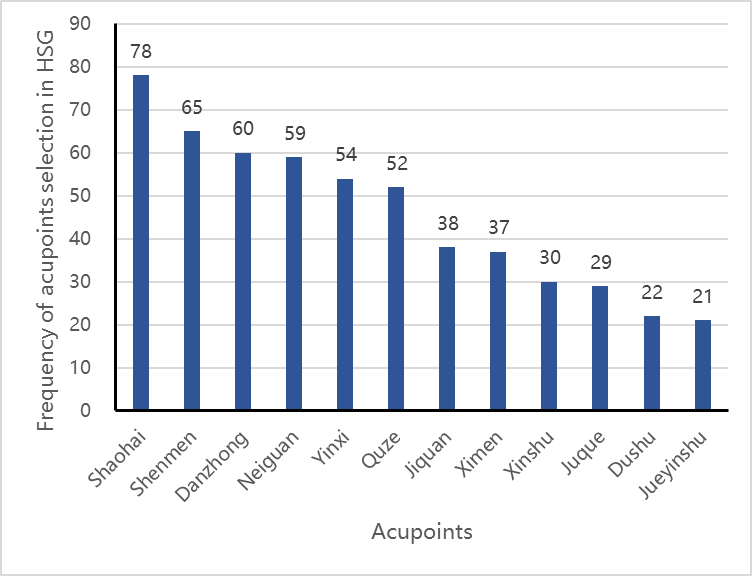


**Figure s3. Frequency of acupoint selection in HSG**

HSG: High-sensitivity group


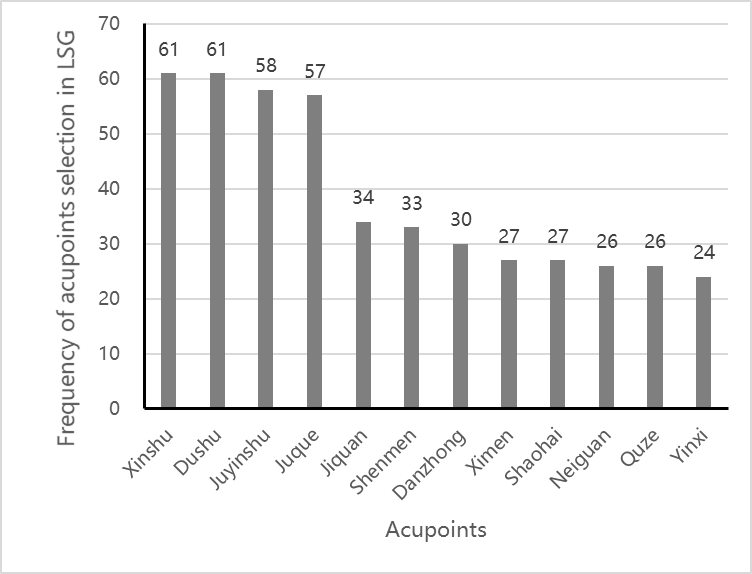


**Figure s4. Frequency of acupoint selection in LSG**

LSG: Low-sensitivity group

**S5 PPT of 12 acupoints at baseline**


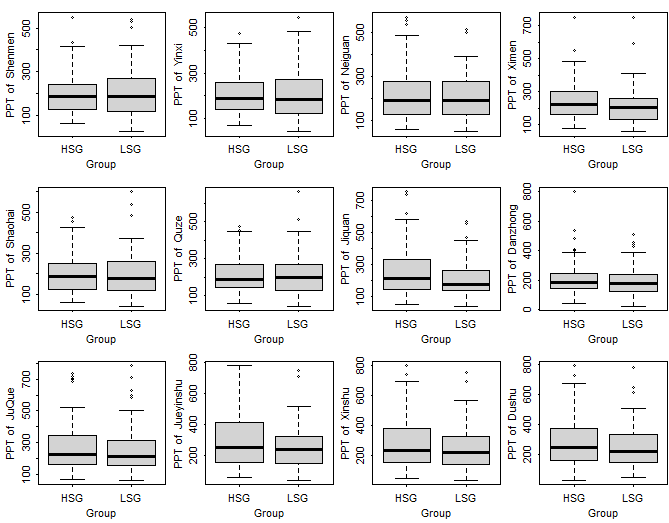


**Figure s5. Frequency of acupoint selection in LSG**

PPT: Pressure-Pain Threshold

**S6 Baseline characteristics of excluded patients**

**Table s1. Baseline characteristics of excluded patients**

| **Characteristic^1^** | **LSG (N = 6)** | **HSG (N = 6)** | **Overall (N = 12)** |
| --- | --- | --- | --- |
| **Age (Mean ± SD)** | 68.50±7.97 | 63.83±9.02 | 66.17±8.47 |
| **Sex (Female)** | 2 (33.3%) | 5 (83.3%) | 7 (58.3%) |
| **Marital status (Married)** | 6 (100.0%) | 6 (100.0%) | 12 (100.0%) |
| **BMI (Mean ± SD)** | 21.05±2.66 | 24.01±4.78 | 22.53±4.00 |
| **Education** |  |  |  |
| Illiteracy | 0 (0.0%) | 0 (0.0%) | 0 (0.0%) |
| Primary school | 2 (33.3%) | 2 (33.3%) | 4 (33.3%) |
| High school | 4 (66.7%) | 3 (50.0%) | 7 (58.3%) |
| University and above | 0 (0.0%) | 1 (16.7%) | 1 (8.3%) |
| **Regular Smoke (Yes)** | 2 (33.3%) | 2 (33.3%) | 4 (33.3%) |
| **Regular Drink (Yes)** | 1 (16.7%) | 1 (16.7%) | 2 (16.7%) |
| **Drug Sensitive History (Yes)** | 0 (0.0%) | 0 (0.0%) | 0 (0.0%) |
| **CHD Family History (Yes)** | 0 (0.0%) | 0 (0.0%) | 0 (0.0%) |
| **Hypertension (Yes)** | 4 (66.7%) | 4 (66.7%) | 8 (66.7%) |
| **Diabetes (Yes)** | 1 (16.7%) | 2 (33.3%) | 3 (25.0%) |
| **Aspirin (Yes)** | 5 (83.3%) | 3 (50.0%) | 8 (66.7%) |
| **Clopidogrel (Yes)** | 0 (0.0%) | 1 (16.7%) | 1 (8.3%) |
| **β-blockers (Yes)** | 3 (50.0%) | 2 (33.3%) | 5 (41.7%) |
| **Statins (Yes)** | 5 (83.3%) | 3 (50.0%) | 8 (66.7%) |
| **Angiotensin Inhibitor (Yes)** | 0 (0.0%) | 0 (0.0%) | 0 (0.0%) |
| **Calcium antagonists (Yes)** | 2 (33.3%) | 3 (50.0%) | 5 (41.7%) |
| **Frequency of Angina Attacks**  **(Mean ± SD)** | 6.83±7.25 | 5.83±3.43 | 6.33±5.43 |
| **Nitroglycerin Consumption** |  |  |  |
| No Use | 3 (50.0%) | 5 (83.3%) | 8 (75.0%) |
| < 3 times/week | 2 (33.3%) | 1 (16.7%) | 3 (25.0%) |
| 3~12 times/week | 1 (16.7%) | 0 (0.0%) | 1 (8.3%) |
| ≥ 12 times/week | 0 (0.0%) | 0 (0.0%) | 0 (0.0%) |
| **CCS Grade** |  |  |  |
| Level I | 4 (66.7%) | 3 (50.0%) | 7 (58.3%) |
| Level II | 1 (16.7%) | 3(50.0%) | 4 (33.3%) |
| Level III | 1 (16.7%) | 0 (0.0%) | 1 (8.3%) |
| Level IV | 0 (0.0%) | 0 (0.0%) | 0 (0.0%) |
| **SAQ score** |  |  |  |
| Physical Limitations | 65.56±15.95 | 72.22±14.86 | 68.89±15.10 |
| Angina Stability | 29.17±33.23 | 29.17±33.23 | 29.17±31.68 |
| Angina Frequency | 60.00±18.97 | 57.92±17.49 | 58.96±17.43 |
| Treatment Satisfaction | 70.59±19.33 | 58.82±9.84 | 64.71±15.86 |
| Quality of Life | 65.28±27.60 | 59.72±22.00 | 62.50±23.97 |

SD: Standard Difference; CHD: Coronary Heart Disease; CCS: Canadian Cardiovascular Society; SAQ: Seattle Angina Questionnaire; ^1^Mean ± SD or Frequency (%); ^2^Wilcoxon rank sum test; Pearson's Chi-squared test; Fisher's exact test

**S7 Mixed effect model of SAQ score of patients in different periods**

**Table s2. Mixed effect model of SAQ score of patients in different periods**

| **Subscale of SAQ score** | **MD** | **95%CI** | ***P-value*** |
| --- | --- | --- | --- |
| Physical Limitations | | | |
| Treatment：HSG vs LSG | -0.5041 | (-2.993,1.984) | 0.346 |
| Follow-up：HSG vs LSG | 0.4838 | (-2.005,2.972) | 0.352 |
| Angina Stability | | | |
| Treatment：HSG vs LSG | 5.9162 | (-1.687,13.519) | 0.064 |
| Follow-up：HSG vs LSG | -0.8198 | (-8.422,6.783) | 0.416 |
| Angina Frequency | | | |
| Treatment：HSG vs LSG | 1.910 | (-1.225,5.045) | 0.116 |
| Follow-up：HSG vs LSG | 3.807 | (0.673,6.942) | 0.009^*^ |
| Treatment Satisfaction | | | |
| Treatment：HSG vs LSG | 3.651 | (0.307, 6.995) | 0.017^*^ |
| Follow-up：HSG vs LSG | 4.220 | (0.876,7.563) | 0.007^*^ |
| Quality of Life | | | |
| Treatment：HSG vs LSG | -2.271 | (-6.472,1.931) | 0.145 |
| Follow-up：HSG vs LSG | 1.341 | (-2.861,5.543) | 0.266 |

SAQ: Seattle Angina Questionnaire; HSG: High-sensitivity group; LSG: Low-sensitivity group; MD: Mean Difference; CI: Confidence Interval; *：P<0.05

**S8 Mixed effect models based on ordinal regression of CCS grade**

**Table s3. Mixed effect model of CCS grade of patients in different periods**

| **Effect** | **Parameters** | **OR (95%CI)** | | |
| --- | --- | --- | --- | --- |
|  |  | **Model A** | **Model B** | **Final Model** |
| Fixed Effect | β_1_ (HSG) | 2.349 (0.405,1.363) | 2.721 (0.356,2.114) | 2.638 (0.305,2.286) |
|  | β_2_ (Week 4) | - | 0.231 (0.110,4.860) | 0.259 (0.091,7.400) |
|  | β_3_ (Week 8) | - | 0.155 (0.071,3.390) | 0.121 (0.039,3.700) |
|  | β_4_ (HSG*Week4) | - | - | 0.805 (0.201,3.222) |
|  | β_5_ (HSG*Week8) | - | - | 1.542 (0.376,6.334) |
| Randomized Effect | σ^2^_u0_ (Center) | 7.892^*^ | 10.28^*^ | 10.33^*^ |
|  | σ^2^_0_ (Individual) | 25.466^*^ | 33.39^*^ | 33.65^*^ |
| Model Fitting | -2loglikelihoods | 701.96 | 672.1 | 671.3 |

CCS: Canadian Cardiovascular Society; HSG: High-sensitivity group; LSG: Low-sensitivity group; OR: Odds Ratio; CI: Confidence Interval
